# ERGs on the brain: the benefits of simultaneous flash retinal and cortical responses in paediatric cerebral visual impairment

**DOI:** 10.1007/s10633-018-9631-4

**Published:** 2018-05-03

**Authors:** Sian E. Handley, Dorothy A. Thompson, Katrina L. Prise, Alki Liasis

**Affiliations:** 1 Clinical and Academic Department of Ophthalmology, Great Ormond Street Hospital for Children, Great Ormond Street, London, WC1N 3JH UK; 2 UCL Great Ormond Street Institute of Child Health, University College London, 30 Guildford Street, London, WC1N 1EH UK

**Keywords:** Cerebral visual impairment (CVI), Flash visual evoked potential (VEP), Flash electroretinogram (ERG), Skin electrodes, Paediatric

## Abstract

**Purpose:**

To highlight the importance of simultaneous flash electroretinogram (ERG) and visual evoked potential (VEP) recording to differentiate a true flash VEP response from an artefact caused by the intrusion of the ERG on a mid-frontal reference electrode in cases of severe cerebral visual impairment (CVI).

**Methods:**

We report an observational case series of four children with severe CVI who underwent simultaneous flash ERG and VEP recordings. Flash VEPs from Oz–Fz and lower lid skin ERGs referred to Fz were recorded simultaneously to Grass intensity setting 4 flash stimulation.

**Results:**

In all cases, atypical, but reproducible VEPs were evident. Comparison of the timing and waveform of the VEPs and ERGs showed the occipital responses were inverted ERGs and no true flash VEP was evident.

**Conclusions:**

While ISCEV and neurophysiology standards do not require the simultaneous recording of the flash ERG with the VEP, these cases highlight the usefulness of this non-invasive technique particularly in suspected paediatric cerebral visual impairment to differentiate a true VEP from an artefact caused by ERG contamination.

## Introduction

In children with severe cerebral visual impairment (CVI), behavioural measures of visual function are rarely obtainable. As a result, visual evoked potentials (VEPs) have become a well-recognised and valuable tool in the assessment of visual pathway function in these children. Pattern VEPs are able to provide an indication of macular pathway function and an estimate of visual potential [1]. In the absence of pattern VEPs, flash VEPs are able to provide a measure of generalised post-retinal activation and determine the presence or absence of chiasmal/hemisphere dysfunction. In children with CVI, identifying the presence or absence of visual pathway activation to the striate cortex has consequences upon the type of support and rehabilitation the patient receives and ultimately on their longer-term quality of life [2].

The typical flash VEP has several negative and positive waves; the most consistent and robust of these are N2 at around 90 ms and P2 at around 120 ms [3].

Though very reproducible within a subject, flash VEPs are highly variable in morphology between individuals [3, 4] making interpretation of atypical responses in the presence of gross pathology challenging. In children with significant CVI, the flash VEPs may be maximally localised to non-standard regions of the scalp [5], atypical in morphology, but also markedly reduced in amplitude or increased in latency [6].

The ability to record responses in the microvolt range relies on the use of differential amplifiers. These only amplify the difference between the input of the active and reference electrodes, thereby improving the signal-to-noise ratio. Although the ideal hypothetical reference for a VEP recording would have zero activity in clinical practice, the reference can be influenced by the background electroencephalogram and environmental artefacts. Apart from artefacts such as muscle confounding the reference site, it can be influenced by activity from atypically distributed visual cortex or by other physiological responses time locked to the flash stimulus [7, 8]. One artefact that has been observed in brain dead patients is the reference contamination by the spread of the electroretinogram (ERG) [9]. One study has demonstrated that the ERG can spread as far back as rolandic fissure [8], while other studies have demonstrated it can be at times detected as far as the occipital area [9].

The purpose of this case series was to demonstrate in the awake child with severe CVI how an absent flash VEP confounded by reference contamination can potentially be misinterpreted in the absence of simultaneously recorded ERGs.

## Methods

We retrospectively identified four children with severe CVI seen within the last year of our clinical practice at the Tony Kriss Visual Electrophysiology Unit, Great Ormond Street Hospital for Children, London, UK, in whom an artefact was evident in the flash VEP making it difficult to identify a true response. Case history, referral details, VEP and ERG waveforms are given for each patient.

### Stimuli and procedure

All patients underwent both eyes open simultaneous flash VEP and ERG testing as per the department protocol. VEP responses were recorded from silver/silver-chloride scalp electrodes placed at O1, O2 and Oz. Skin ERG electrodes were placed on the inferior orbital rim close to the lower eyelid. All electrodes were referred to Fz, while the ground was placed at POz. Impedance was maintained at below 5 kΩ, and all recordings were undertaken in mesopic conditions. A handheld strobe (Grass model PS22) was used to present the flash stimuli at an intensity setting 4 and a stimulation rate of 3 flashes/second (3 Hz). A minimum of two trials were recorded to each stimulus before a grand average was created.

### Patient 1

An 8-years-old attended the department for the evaluation of vision loss. Vision had been normal, until a few months previously when they had an intraoperative collapse following induction aesthetic for routine surgery. Post-operatively the visual acuity was reported as no perception of light in both eyes and roving eye movements seen. Fundus and media examination revealed normal retina appearance and bilaterally pale and atrophic optic nerves with paradoxical pupils.

### Patient 2

A 15-years-old with neurofibromatosis type 1 and bilateral optic pathway gliomas was referred for review prior to starting a palliative drug trial as the tumours had been resistant to conventional glioma chemotherapy treatment protocols, and all other treatment options had been exhausted. The best-corrected visual acuity in the right eye was 1.7 logMAR and in the left eye hand movements. Fundoscopy revealed severe bilateral optic atrophy. Manifest rotary nystagmus was evident.

### Patient 3

A 1-month-old infant was transferred to neonatal intensive care with apnoea and seizures. Examination findings showed bilateral frontoparietal polymicrogyria, neuronal migration disorder, microcephaly and dysmorphism. He had an ophthalmology review at 7 months of age because the infant was not fixing and following. Ocular examination showed normal anterior segment, fundi and media. Visual electrophysiology testing was requested to assess visual potential, which was carried out at age 10 months.

### Patient 4

A 4-years-old was referred to the department for the assessment of visual function as part of medico-legal proceedings. The child had suffered hypoxic ischaemic encephalopathy during a traumatic birth and as a result spastic quadriplegia and seizure disorder. On clinical examination, the child had a right divergent squint with no demonstrable fix and follow. Fundoscopy showed normal retinal and foveal appearances with bilateral optic atrophy.

## Results

**Fig. 1:**
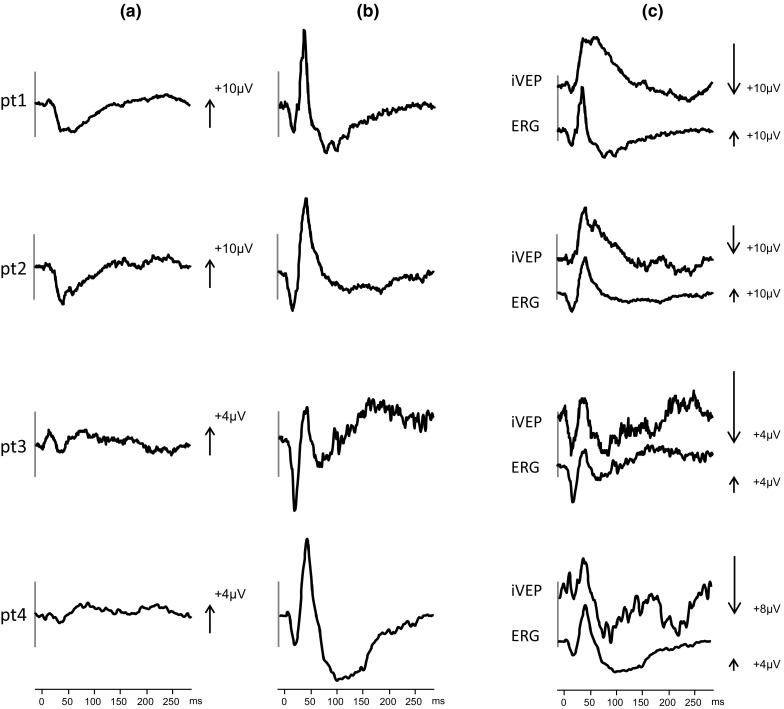
The flash VEP (**a**) and ERG (**b**) responses simultaneously obtained from all patients (pt1–4). The inverted VEP (iVEP) is shown above the ERG scaled for visual comparison of waveforms (**c**)

In all cases, responses were recorded from O1, O2 and Oz referred to Fz. The responses were atypical in morphology for a flash VEP and consisted of an early positive–negative–positive complex (Fig. 1a). An identical waveform was seen from all active channels. In patient 2, monocular flash VEP testing was also carried out and a waveform identical to that seen both eyes open was evident for either eye but a third of the amplitude.

The simultaneously recorded ERG responses are shown for comparison (Fig. 1b). Further inspection of the timing and waveform of the VEP responses revealed that these were inverted ERGs. This was most obvious when the VEP response was inverted (iVEP) and compared to the ERG (Fig. 1c).

**Fig. 2:**
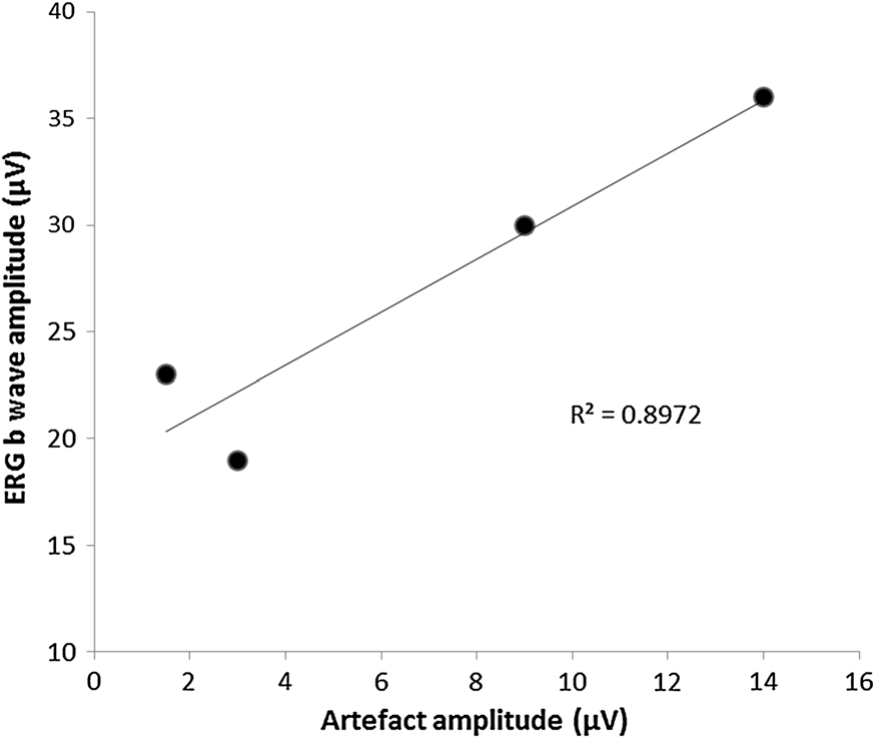
The amplitude of the ERG b-wave compared to the amplitude of the artefact seen in the VEP shows a linear relationship

Comparison of the simultaneously recorded responses between cases indicated a relationship between the amplitude of the ERG recorded and the artefact revealed in the VEP [*R*^2^ = 0.8972] (Fig. 2). Patients 1 and 2 had larger amplitude skin ERGs (30 and 36 µV b-waves) resulting in larger responses in the VEP channel compared to patients 3 and 4 (ERG b-waves 19 and 23 µV). All of the ERG b-wave amplitudes were within the laboratory’s normative values for age.

## Discussion

The flash VEP is a highly feasible and valuable tool in the assessment of visual pathway function in children with CVI, particularly when behavioural testing is limited [10–12]. These cases illustrate how in children with extremely marked CVI a flash VEP can be contaminated by retinal activity recorded by the reference electrode. As a result, it can be difficult to distinguish a true VEP response in isolation without recording an ERG for comparison.

The location of the mid-frontal reference (Fz) is susceptible to the contamination from ERG field spread [8, 9]. In an attempt to overcome this problem of reference electrode, contamination alternative reference electrode sites have been investigated. Ear lobe and nose references have been shown to also be susceptible to ERG contamination [7, 9]. Non-cephalic references such as an inverted electrocardiogram have been suggested; however, commercially available visual electrophysiology recording systems are not designed for this and even with a non-cephalic reference the ERG has been demonstrated to spread to the active occipital electrodes [9, 13].

The use of concurrent skin ERG recordings provides an easy control marker of reference contamination. The International Society of Clinical Electrophysiology of Vision (ISCEV) and International Federation of Clinical Neurophysiology (IFCN) as a minimum standard do not suggest a flash ERG to be performed simultaneously during a flash VEP in CVI [3, 14]. However, the ISCEV standards for a flash VEP do require the flash strength to be within the same limits as those used for the standard mixed rod/cone flash ERG [3], making concurrent recording possible and feasible.

Skin electrodes are used for ERG recording in paediatric electrophysiology as they are better tolerated by alert infants and children than corneal electrodes used in adult practice [15, 16] and obviate a need for sedation or anaesthesia. In children, skin ERG electrodes can be easily applied and add minimal testing time. The convincing diagnostic advantages of recording the VEP concurrently with the skin ERG in young children to identify other recording artefacts that may occur have previously been discussed [15]. As well as differentiating the ERG artefact from a true response, they also provide a control that these cases were all adequately stimulated with the light in the presence of the absent flash VEP. This is particularly useful in paediatric work where co-operation of the patient can be unpredictable.

This ERG artefact is rarely noticed when a typical VEP is present as it is proportionally very small in amplitude compared to a normal flash VEP. However, when it occurs in marked pathway dysfunction, the ERG is relatively large compared to the near isoelectric background response recoded at Oz. In this situation, it is difficult to differentiate evidence of real post-retinal activation, especially as the inverted polarity descending limb of the b-wave extends into the 100–150 ms range where you would typically look for the P2 component of the flash VEP.

## Conclusions

The flash VEP is highly useful in the assessment of visual pathway function in marked paediatric CVI. The implications of identifying post-retinal activation in these cases are often weighty. Adding skin ERG electrodes to paediatric flash VEP recording protocols is a simple, but highly effective control measure to monitor the quality of the recordings obtained. This control measure allows the differentiation of artefacts caused by ERG contamination from true post-retinal activation in cases of severe CVI.

## References

[1] Weiss AH, Kelly JP, Phillips JO (2001). The infant who is visually unresponsive on a cortical basis. Ophthalmology.

[2] Hoyt CS (2007). Brain injury and the eye. Eye.

[3] Odom JV, Bach M, Brigell M (2016). ISCEV STANDARDS ISCEV standard for clinical visual evoked potentials: (2016 update). Doc Ophthalmol.

[4] Halliday A (1992). Evoked potentials in clinical testing.

[5] Handley SE, Liasis AC (2017). Multichannel visual evoked potentials in the assessment of visual pathways in children with marked brain abnormalities. J AAPOS.

[6] Kuba M, Liláková D, Hejcmanová D (2008). Ophthalmological examination and VEPs in preterm children with perinatal CNS involvement. Doc Ophthalmol.

[7] Herr DW, Vo KT, King D, Boyes WK (1996). Possible confounding effects of strobe “clicks” on flash evoked potentials in rats. Physiol Behav.

[8] Heckenlively JR, Arden GB (1991). Principles and practice of clinical electrophysiology of vision.

[9] Machado C, Santiesteban R, García O (1993). Visual evoked potentials and electroretinography in brain-dead patients. Doc Ophthalmol.

[10] McCulloch DL, Taylor MJ (1992). Cortical blindness in children: utility of flash VEPs. Pediatr Neurol.

[11] Taylor MJ, McCulloch DL (1991). Prognostic value of VEPs in young children with acute onset of cortical blindness. Pediatr Neurol.

[12] Frank Y, Kurtzberg D, Kreuzer JA, Vaughan HG (1992). Flash and pattern-reversal visual evoked potential abnormalities in infants and children with cerebral blindness. Dev Med Child Neurol.

[13] Nakamura M, Shibasaki H, Nishida S (1990). Method for recording short latency evoked potentials using an EKG artifact elimination procedure. J Biomed Eng.

[14] Holder GE, Celesia GG, Miyake Y (2010). International federation of clinical neurophysiology: recommendations for visual system testing. Clin Neurophysiol.

[15] Kriss A (1994). Skin ERGs: their effectiveness in paediatric visual assessment, confounding factors, and comparison with ERGs recorded using various types of corneal electrode. Int J Psychophysiol.

[16] Fulton AB, Brecelj J, Lorenz B (2006). Pediatric clinical visual electrophysiology: a survey of actual practice. Doc Ophthalmol.

